# Ciprofloxacin-Induced Unilateral Tremor and Weakness Mimicking Acute Ischemic Stroke: A Case Report

**DOI:** 10.7759/cureus.103854

**Published:** 2026-02-18

**Authors:** Abhigyan Dwivedi, Arshad Hussain

**Affiliations:** 1 Medicine, Ipswich Hospital - West Moreton Health, Brisbane, AUS

**Keywords:** adult neurology, medication side-effects, stroke protocol, stroke systems of care, stroke mimic

## Abstract

Fluoroquinolones are widely prescribed antibiotics and are generally well-tolerated; however, they are associated with rare but clinically significant neurological adverse effects that may mimic acute cerebrovascular events.

We describe the case of a 50-year-old man who developed intermittent unilateral coarse tremors and weakness of the left upper limb approximately two weeks after initiating ciprofloxacin therapy following endoscopic retrograde cholangiopancreatography (ERCP) for suspected choledocholithiasis. The acute presentation prompted a Code Stroke activation. Initial computed tomography (CT) of the brain and CT angiography (CTA) of the head and neck showed no evidence of acute infarction or large vessel occlusion. Subsequent magnetic resonance imaging (MRI) of the brain with diffusion-weighted imaging definitively excluded acute or subacute ischemia.

Ciprofloxacin was discontinued due to suspicion of drug-induced neurotoxicity, and the patient’s neurological symptoms resolved completely within 48 hours. Follow-up at one month demonstrated sustained recovery and absence of recurrence.

This case highlights ciprofloxacin-induced neurotoxicity, an important stroke mimic with a Naranjo Adverse Drug Reaction Probability Scale score of 7, and, as such, underscores the importance of thorough medication reconciliation in patients presenting with acute focal neurological deficits.

## Introduction

Fluoroquinolones, including ciprofloxacin, are commonly used broad-spectrum antibiotics with excellent tissue penetration, including the central nervous system [1]. While generally safe, they have been associated with neurological adverse effects such as tremor, myoclonus, seizures, and neuropsychiatric symptoms [1-3]. These effects are thought to occur through antagonism of gamma-aminobutyric acid (GABA) receptors, leading to neuronal hyperexcitability [2].

Acute focal neurological deficits are typically managed as presumed ischaemic stroke until proven otherwise. However, drug-induced neurological syndromes may closely resemble cerebrovascular events, creating diagnostic uncertainty and exposing patients to potentially unnecessary investigations and treatment [3]. Unilateral tremor with focal limb weakness as a manifestation of ciprofloxacin-induced neurotoxicity remains rarely reported. This case highlights the importance of careful medication review in patients presenting with suspected acute stroke.

## Case presentation

A 50-year-old male truck driver presented to the Emergency Department at Ipswich Hospital, Queensland, Australia, with acute-onset intermittent involuntary movements and weakness affecting the left upper limb. Two weeks prior to presentation, he had been commenced on ciprofloxacin 500 mg twice daily following an admission in New South Wales for cholelithiasis with suspected choledocholithiasis, during which he underwent endoscopic retrograde cholangiopancreatography.

His past medical history was significant for chronic obstructive pulmonary disease, gastroesophageal reflux disease, and an umbilical hernia. These conditions were managed conservatively in the community, and he was not taking any regular neuroactive or centrally acting medications. He had no history of renal impairment, and the neurological symptoms developed approximately 12 days after initiation of ciprofloxacin therapy. He had no prior history of cerebrovascular disease, seizure disorder, or movement disorders. He was an active smoker with an approximately 30-pack-year history and denied alcohol consumption or illicit drug use.

Symptoms began the evening prior to presentation with intermittent episodes of rhythmic, coarse tremors predominantly involving the left wrist. These episodes occurred both at rest and with movement, lasted several minutes, and were associated with paraesthesia and subjective weakness of the same limb. By the following morning, the patient noted worsening weakness with difficulty extending the wrist, prompting hospital presentation and activation of the Code Stroke pathway.

On initial assessment, vital signs were stable. Neurological examination demonstrated a National Institutes of Health Stroke Scale (NIHSS) score of 2, attributable solely to left upper limb motor weakness. Cranial nerve examination was normal, with no facial asymmetry, dysphasia, visual disturbance, or sensory deficit.

On subsequent medical review, the tremor episodes were not observed. Motor examination revealed persistent left upper limb weakness graded 4/5 across C5-C8 myotomes. Tone, coordination, and deep tendon reflexes were normal, with no spasticity or rigidity. Examination of the right upper limb and both lower limbs was unremarkable.

Investigations

Laboratory investigations are summarised in Table 1. These demonstrated preserved renal function, mild leukocytosis, and a resolving mixed cholestatic and hepatocellular pattern consistent with recent biliary obstruction. Electrolytes, coagulation studies, lactate, thyroid function, and glycaemic indices were within normal limits.

**Table 1:** Baseline laboratory investigations at presentation eGFR = estimated glomerular filtration rate; ALT = alanine aminotransferase; AST = aspartate aminotransferase; GGT = gamma-glutamyl transferase; FT4 = free thyroxine; HbA1c = glycated haemoglobin; HDL = high-density lipoprotein

Parameters	Patient Values	Reference Range
Haemoglobin	156 g/L	130–180 g/L
White cell count	11.7 ×10⁹/L	4.0–11.0 ×10⁹/L
Platelet count	225 ×10⁹/L	150–400 ×10⁹/L
Sodium	141–142 mmol/L	135–145 mmol/L
Potassium	4.0–4.7 mmol/L	3.5–5.2 mmol/L
Chloride	103–107 mmol/L	95–110 mmol/L
Bicarbonate	28–30 mmol/L	22–32 mmol/L
Creatinine	69–81 µmol/L	60–110 µmol/L
Estimated GFR	>90 mL/min/1.73 m²	>60 mL/min/1.73 m²
Total bilirubin	29 µmol/L	<21 µmol/L
Conjugated bilirubin	20–21 µmol/L	<5 µmol/L
Alkaline phosphatase	120–122 U/L	30–110 U/L
Gamma-glutamyl transferase	251–285 U/L	<60 U/L
Alanine aminotransferase	113–129 U/L	<45 U/L
Aspartate aminotransferase	48–50 U/L	<40 U/L
Lactate	1.2 mmol/L	0.5–2.2 mmol/L
Thyroid-stimulating hormone	1.06 mIU/L	0.4–4.0 mIU/L
Free thyroxine (FT4)	14.6 pmol/L	9–19 pmol/L
HbA1c	5.2%	<5.7%
Triglycerides	1.8 mmol/L	<1.7 mmol/L
HDL cholesterol	0.5 mmol/L	>1.0 mmol/L

**Figure 1:**
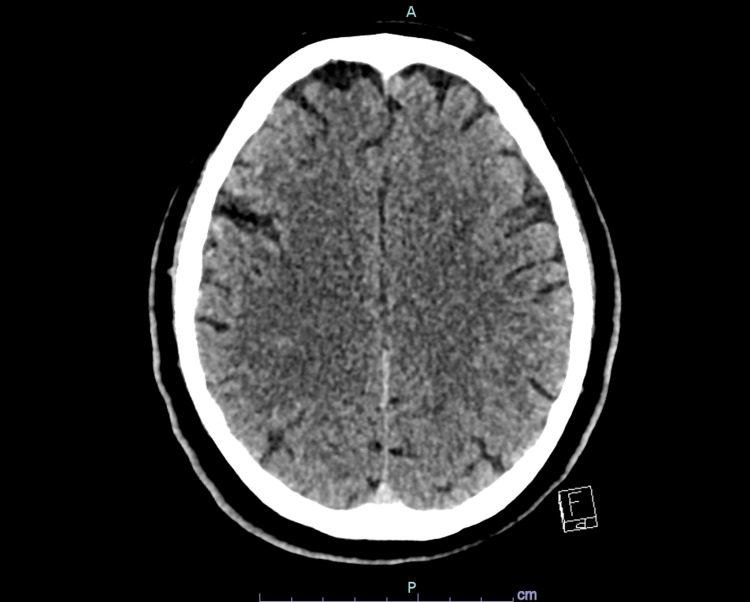
Non-contrast axial CT brain at a high convexity level demonstrating no acute intracranial haemorrhage or infarction

**Figure 2:**
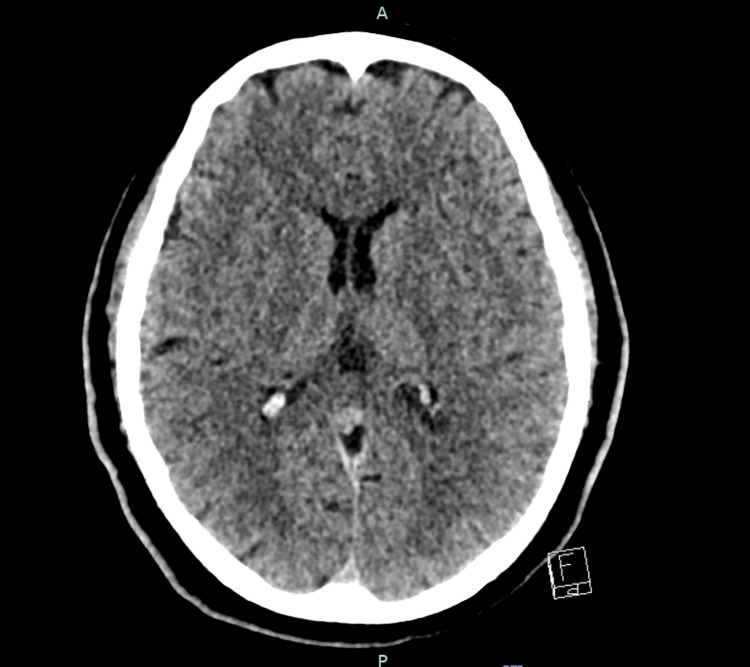
Non-contrast axial CT brain at the level of the basal ganglia demonstrating no acute intracranial abnormality

**Figure 3:**
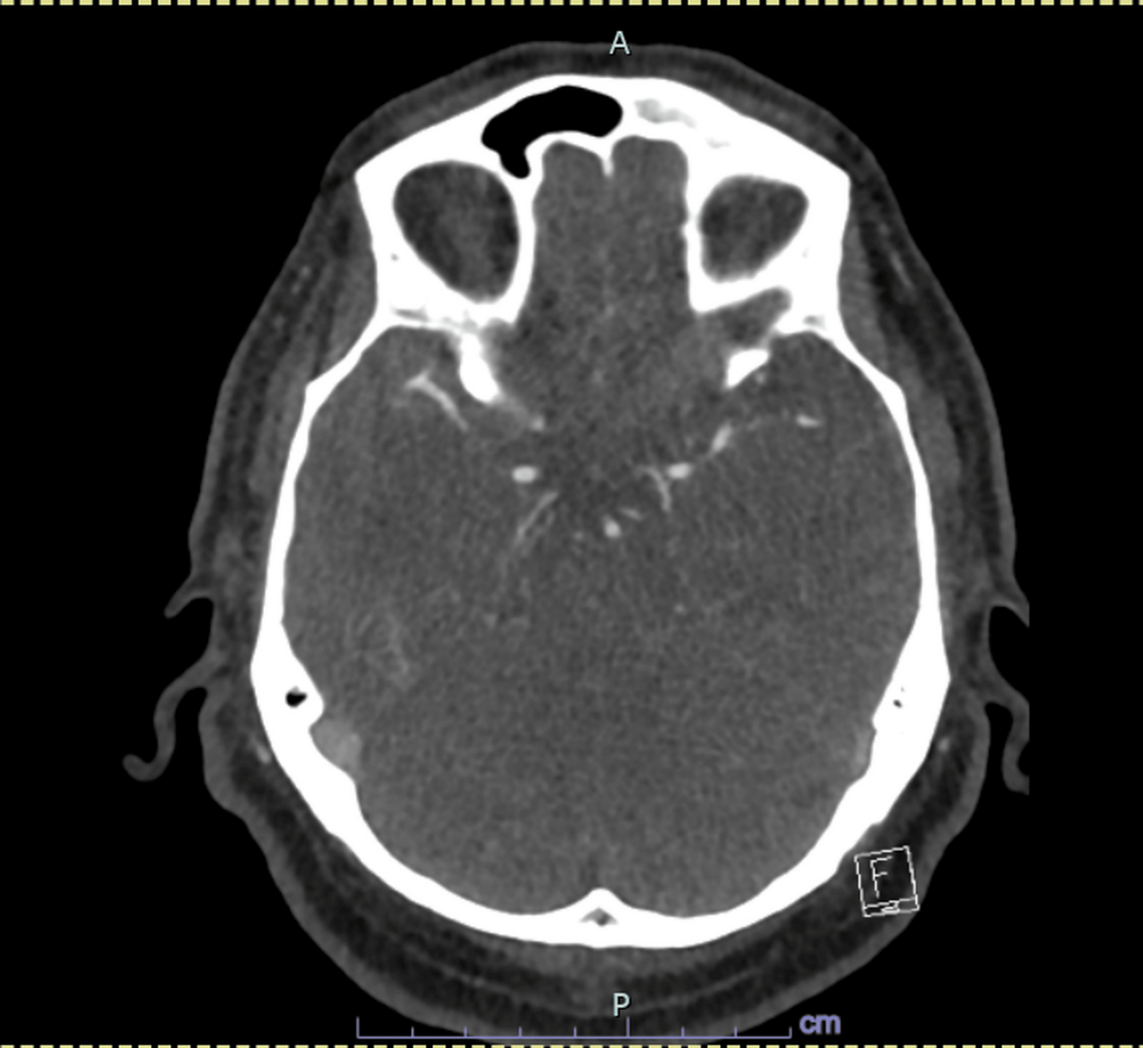
Axial CT angiography of the head at the level of the Circle of Willis demonstrating patent intracranial arteries with no evidence of large vessel occlusion

**Figure 4:**
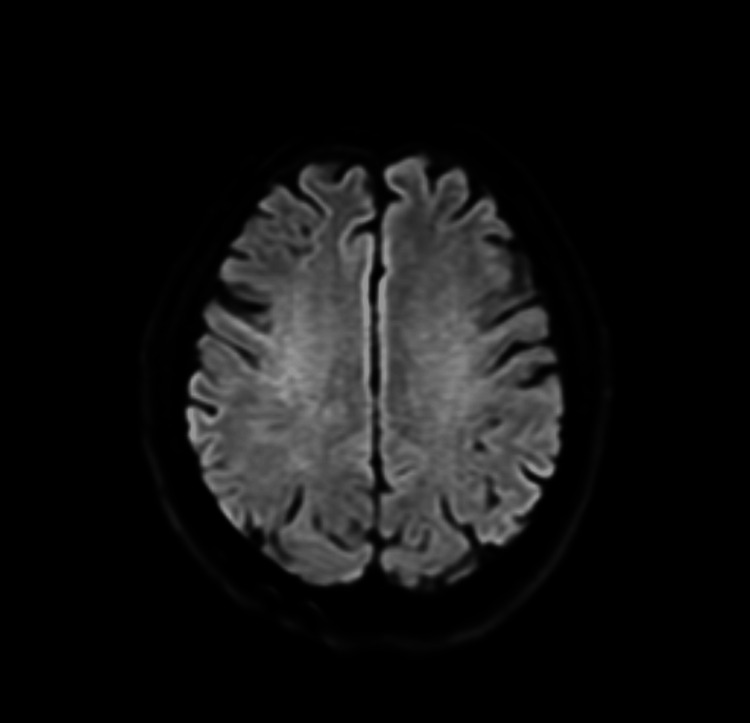
Axial diffusion-weighted MRI of the brain at the level of the centrum semiovale demonstrating no diffusion restriction

**Figure 5:**
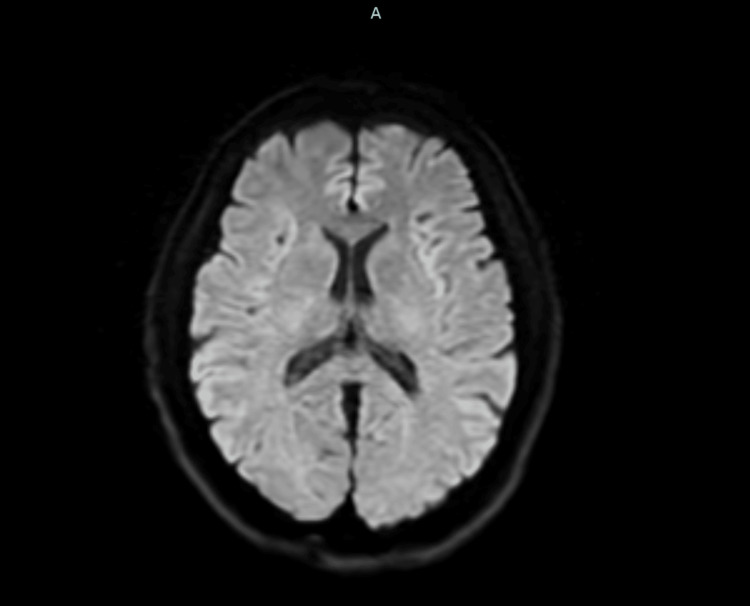
Axial diffusion-weighted MRI of the brain at the level of the basal ganglia demonstrating no diffusion restriction

Urgent non-contrast computed tomography (CT) of the brain demonstrated no evidence of acute intracranial haemorrhage or established infarction (Figures 1, 2). CT angiography of the head showed patent intracranial arteries with no evidence of large vessel occlusion (Figure 3). Magnetic resonance imaging (MRI) of the brain with diffusion-weighted imaging was subsequently performed and demonstrated no diffusion restriction or structural abnormality, effectively excluding acute or subacute ischaemic stroke (Figures 4, 5). 

Differential diagnosis

The initial differential diagnosis included acute ischaemic stroke or transient ischaemic attack, given the sudden onset of unilateral weakness. Todd’s paresis following a focal seizure was considered due to the episodic nature of the involuntary movements. Drug-induced neurotoxicity, particularly fluoroquinolone-associated neurological adverse effects, was suspected given the clear temporal relationship with ciprofloxacin initiation [1-3]. An intracranial structural lesion was considered less likely and was subsequently excluded through comprehensive neuroimaging.

Treatment

The patient was initially managed according to acute stroke protocol and commenced on dual antiplatelet therapy and high-dose atorvastatin while awaiting definitive neuroimaging.

Following MRI confirmation of the absence of cerebral ischaemia on day three and review of the medication history, ciprofloxacin was discontinued and replaced with oral amoxicillin-clavulanate. Dual antiplatelet therapy and high-dose atorvastatin were also ceased on day three.

Outcome and follow-up

Following cessation of ciprofloxacin, the patient’s tremor episodes ceased completely, and the residual left upper limb weakness resolved over the subsequent 24-48 hours. Follow-up at one month demonstrated sustained recovery with the absence of recurrence of the tremor and return to baseline power of the left upper limb. Dual antiplatelet therapy was discontinued after ischaemic stroke was definitively excluded. The patient was discharged home neurologically intact.

Application of the Naranjo Adverse Drug Reaction Probability Scale yielded a score of 7, indicating a probable adverse drug reaction, based on the clear temporal relationship, resolution following drug withdrawal, prior published reports, and exclusion of alternative causes [4].

## Discussion

Compared with previously reported fluoroquinolone-associated movement disorders, this case demonstrates features consistent with the existing literature. Prior reports describe neurotoxicity emerging within days to several weeks after exposure, most commonly presenting with tremor, myoclonus, seizures, or encephalopathy, and often demonstrating rapid reversibility following drug cessation [2,3,5]. In our patient, symptom onset approximately two weeks after initiation and complete resolution within 24-48 hours of withdrawal closely mirror these patterns. Although renal impairment is a recognised risk factor due to reduced drug clearance and accumulation, fluoroquinolone neurotoxicity has also been described in patients with preserved renal function, as observed here [5,6].

The proposed pathophysiology involves competitive antagonism of γ-aminobutyric acid (GABA-A) receptors resulting in neuronal disinhibition, with additional contributions from N-methyl-D-aspartate (NMDA) receptor facilitation and oxidative neuronal stress [7-9]. Experimental studies have demonstrated concentration-dependent inhibition of benzodiazepine-GABA receptor binding by fluoroquinolones, supporting a biologically plausible mechanism for central nervous system hyperexcitability [9]. While most reported cases describe generalised or multifocal manifestations, unilateral presentations, such as in this patient, may reflect focal cortical susceptibility or asymmetric network excitability rather than structural pathology [2,10].

This report has several limitations. Electroencephalography was not performed, and longer-term neurological follow-up beyond one month was not available. However, at the one-month clinical review, the patient remained asymptomatic with no recurrence of tremor or weakness. Causality is based on temporal association, exclusion of alternative etiologies, and Naranjo scoring in a single patient. Nevertheless, the clear dechallenge response and supportive literature strongly implicate ciprofloxacin-induced neurotoxicity as the most likely explanation for this presentation.

## Conclusions

Ciprofloxacin-induced neurotoxicity should be considered in patients presenting with acute focal neurological deficits, particularly when initial imaging is negative and there is recent fluoroquinolone exposure. Early recognition and prompt medication review may prevent unnecessary thrombolysis, antithrombotic therapy, and prolonged hospital admission.
